# Differential Dynamics of the Maternal Immune System in Healthy Pregnancy and Preeclampsia

**DOI:** 10.3389/fimmu.2019.01305

**Published:** 2019-06-11

**Authors:** Xiaoyuan Han, Mohammad S. Ghaemi, Kazuo Ando, Laura S. Peterson, Edward A. Ganio, Amy S. Tsai, Dyani K. Gaudilliere, Ina A. Stelzer, Jakob Einhaus, Basile Bertrand, Natalie Stanley, Anthony Culos, Athena Tanada, Julien Hedou, Eileen S. Tsai, Ramin Fallahzadeh, Ronald J. Wong, Amy E. Judy, Virginia D. Winn, Maurice L. Druzin, Yair J. Blumenfeld, Mark A. Hlatky, Cecele C. Quaintance, Ronald S. Gibbs, Brendan Carvalho, Gary M. Shaw, David K. Stevenson, Martin S. Angst, Nima Aghaeepour, Brice Gaudilliere

**Affiliations:** ^1^Department of Anesthesiology, Perioperative and Pain Medicine, School of Medicine, Stanford University, Palo Alto, CA, United States; ^2^Department of Pediatrics, School of Medicine, Stanford University, Palo Alto, CA, United States; ^3^Department of Surgery, School of Medicine, Stanford University, Palo Alto, CA, United States; ^4^March of Dimes Prematurity Research Center, School of Medicine, Stanford University, Palo Alto, CA, United States; ^5^Department of Obstetrics and Gynecology, School of Medicine, Stanford University, Palo Alto, CA, United States; ^6^Department of Health Research and Policy, School of Medicine, Stanford University, Palo Alto, CA, United States

**Keywords:** preeclampsia, immunology, mass cytometry, PBMC, pregnancy

## Abstract

Preeclampsia is one of the most severe pregnancy complications and a leading cause of maternal death. However, early diagnosis of preeclampsia remains a clinical challenge. Alterations in the normal immune adaptations necessary for the maintenance of a healthy pregnancy are central features of preeclampsia. However, prior analyses primarily focused on the static assessment of select immune cell subsets have provided limited information for the prediction of preeclampsia. Here, we used a high-dimensional mass cytometry immunoassay to characterize the dynamic changes of over 370 immune cell features (including cell distribution and functional responses) in maternal blood during healthy and preeclamptic pregnancies. We found a set of eight cell-specific immune features that accurately identified patients well before the clinical diagnosis of preeclampsia (median area under the curve (AUC) 0.91, interquartile range [0.82–0.92]). Several features recapitulated previously known immune dysfunctions in preeclampsia, such as elevated pro-inflammatory innate immune responses early in pregnancy and impaired regulatory T (Treg) cell signaling. The analysis revealed additional novel immune responses that were strongly associated with, and preceded the onset of preeclampsia, notably abnormal STAT5ab signaling dynamics in CD4^+^T cell subsets (AUC 0.92, *p* = 8.0E-5). These results provide a global readout of the dynamics of the maternal immune system early in pregnancy and lay the groundwork for identifying clinically-relevant immune dysfunctions for the prediction and prevention of preeclampsia.

## Introduction

Preeclampsia is a severe complication of pregnancy defined by the new onset of hypertension and signs of maternal organ dysfunction after the 20th week of gestation (1). Preeclampsia affects between 2 and 8% of all pregnant women—over 8 million women per year worldwide—and is a leading cause of maternal deaths (9–26%) (2). Preeclampsia also accounts for significant neonatal morbidity and mortality due to intrauterine growth restriction, intrauterine fetal demise, and preterm delivery (2). However, no diagnostic test reliably detects preeclampsia early in its development, so treatment can be started only after the onset of signs and symptoms, at which point irreparable harm to the mother and fetus may already have occurred.

Preeclampsia is a multisystem disorder characterized by placental and endothelial dysfunction, leading to hypertension and other end-organ damage such as impaired kidney, liver, neurological, or hematological function (3). Well-described placental abnormalities, including in trophoblast invasion and uterine spiral artery formation, suggest that the roots of preeclampsia are established in the first weeks of pregnancy, before the development of signs and symptoms (4). While markers of placental and endothelial dysfunction—such as increases in soluble FMS-like tyrosine kinase 1 (sFLT-1) levels, and decreases in vascular endothelial growth factor (VEGF) and placental growth factor (PLGF) levels—can be valuable clinically in ruling out suspected preeclampsia (5), early diagnosis of preeclampsia remains clinically challenging.

Systemic inflammation and alterations in the normal immune adaptations necessary for the maintenance of a healthy pregnancy are central features in the pathophysiology of preeclampsia (6–11). Accumulating evidence suggests that preeclampsia is associated with a breakdown of tolerogenic cellular adaptations, including a shift in T cell distributions toward Th1 and Th17 and away from Th2 and regulatory CD4^+^T cell (Treg) populations (12–14). The potential role of the maternal immune system in the pathogenesis of preeclampsia was underscored by a recent multi-omic study of placental, coagulation, complement and vascular factors, highlighting that a majority of plasma proteins associated with preeclampsia were linked to immune functions (15).

Immune dysfunction may be detected well before the clinical onset of preeclampsia, as early as during the first trimester of pregnancy (16–18). For this reason, identifying immunological attributes in maternal blood that predict and help prevent preeclampsia at a preclinical state is of considerable clinical interest (3, 18–20). However, due to limitations in assay technology, prior studies of immune responses associated with preeclampsia have been restricted to a select number of cell subsets and may not have captured immune cell behaviors in the context of the entire peripheral immune system. In particular, the limited number of parameters available for the phenotypic and functional characterization of immune cell subsets may have hampered the detection of important cellular and functional signatures.

Recently developed, highly multiplex single-cell technologies such as mass cytometry—a flow cytometry platform that allows assessment of over 40 parameters on a cell-by-cell basis—offer unprecedented opportunities for comprehensive functional studies of the human immune system (21, 22). Combined with appropriate statistical tools that account for the high-dimensionality of the data, mass cytometry is uniquely capable of identifying alterations of the human immune system associated with normal physiological perturbations and disease pathogenesis (23–25).

In a recent study, we employed a high-parameter mass cytometry assay to characterize the dynamic changes in maternal immune cell distribution and signaling responses during an uncomplicated pregnancy (26). Here, we report on an in-depth profiling of the dynamics of the maternal immune system in healthy (normotensive) pregnancies and preeclampsia. Our primary goal was to detect characteristic immune dysfunctions in the maternal blood prior to the clinical onset of preeclampsia.

## Materials and Methods

### Study Design

Pregnant women participating in a cohort study sponsored by the March of Dimes Prematurity Research Center were prospectively examined for an array of environmental and biological factors associated with uncomplicated and pathological pregnancies (27, 28). Participants all received routine antepartum care at the Lucile Packard Children's Hospital at Stanford University and were eligible for the study if they were 18 years of age or older and in their first trimester of pregnancy. Peripheral blood samples were obtained at least at 2 time points during pregnancy. The study was approved by the Institutional Review Board of Stanford University, and all participants signed an informed consent.

An in-depth mass cytometry analysis of peripheral immune cell responses was performed using the first (median 11 ± 1.9 weeks) and last samples (median 25 ± 4.1 weeks) collected from two subsets of study participants (11 women who developed preeclampsia and 12 women with a normotensive pregnancy). Sample selection criteria for analysis included a cell viability of over 60% and a cell count of over 10^6^ cells. Samples from the 11 women in the preeclampsia group were selected based on a diagnosis of preeclampsia made (and verified by a senior obstetrician) according to the American College of Obstetricians and Gynecologists criteria (1). Early-onset preeclampsia was defined as preeclampsia developing before 34 weeks of gestation (1). Samples from the 12 women in the control group were selected if study participants had a normotensive pregnancy leading to the delivery of a healthy neonate at term (gestational age > 37 weeks), and to ensure matching of gestational age at time of sampling with the preeclampsia group. One patient in the control group had well-managed gestational diabetes mellitus (GDM) with an otherwise uncomplicated pregnancy.

Demographics and pregnancy characteristics for the 23 participants included in the analysis are summarized in Table 1.

**Table 1:** Demographics of study participants.

	**Control** **(*n* = 12)**	**Preeclampsia** **(*n* = 11)**
**DEMOGRAPHICS**		
Age (years, mean ± SD)	33.4 ± 4.7	30.6 ± 5.4
BMI (kg/m^2^, mean ± SD)	24.5 ± 5.6	29.4 ± 4.6 ^*^
BMI at delivery (kg/m^2^, mean ± SD)	28.2 ± 4.7	33.4 ± 4.5 ^*^
GA at delivery (weeks, mean ± SD)	39.3 ± 1.2	37.6 ± 3.0
Gravida (mean ± SD)	3.0 ± 1.5	2.5 ± 2.5
Para (mean ± SD)	1.5 ± 1.5	0.7 ± 1.5
Twin pregnancy	0	1
**RACE/ETHNICITY**		
Asian	0	3
Black	0	1
White	10	4
Other	2	3
Hispanic	3	3
Non-hispanic	9	8
**MODE OF DELIVERY**		
Normal spontaneous vaginal delivery	8	5
Cesarean delivery	4	6
**PREECLAMPSIA CHARACTERISTICS**		
Preeclampsia with severe feature		7
Early-onset preeclampsia		2
**COMORBIDITY**		
Gestational diabetes	1	1
Type II diabetes	0	2
Autoimmune disease	0	3
Chronic hypertension	0	2

* *p < 0.05, by using unpaired student t-test*.

### Sample Collection and PBMC Stimulation

**Figure 1:**
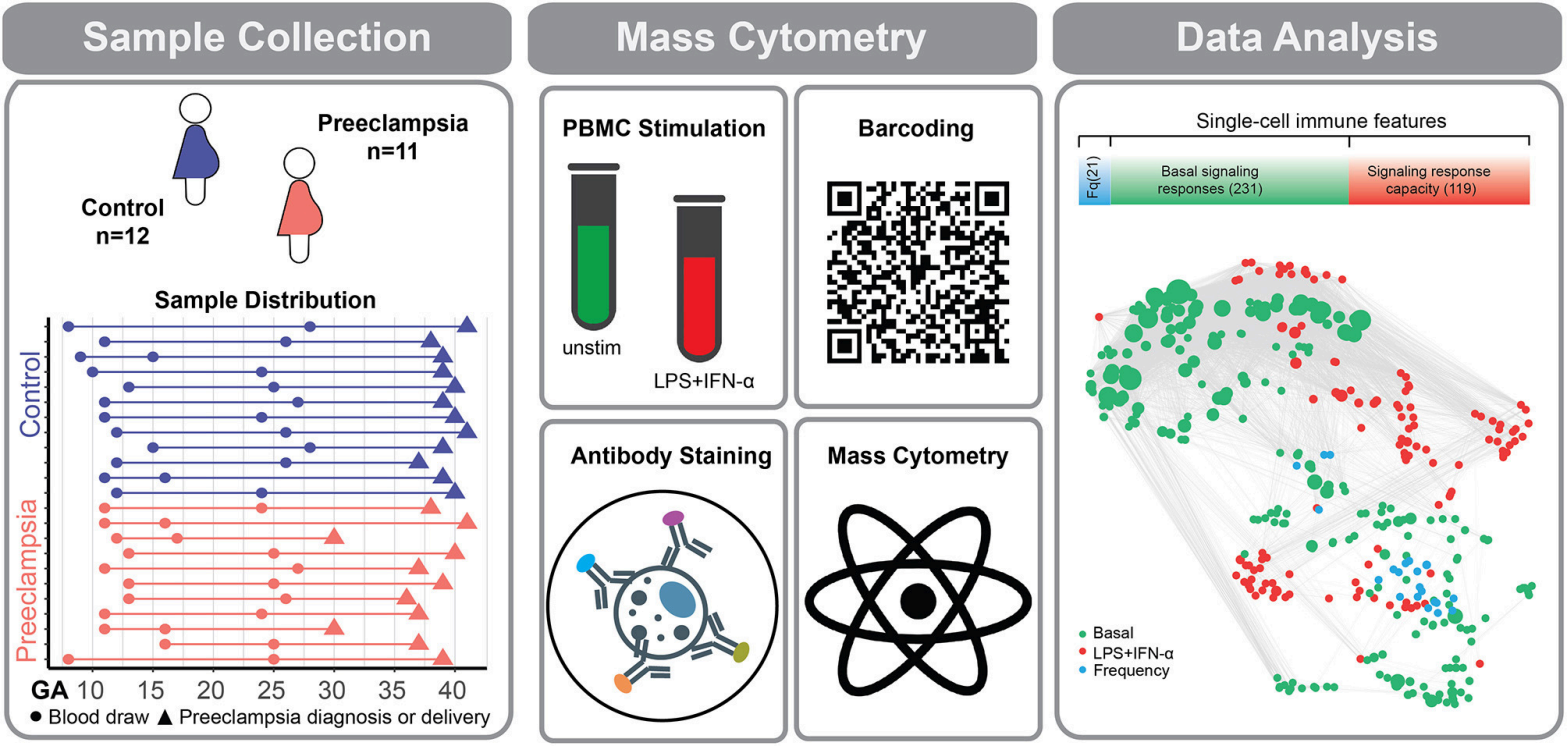
Experimental workflow for the deep profiling of immune system dynamics in preeclampsia. Eleven women with preeclampsia and 12 healthy (normotensive) women were studied. PBMCs were obtained at two time points during the first 28 weeks of pregnancy. Sample collection time (dots), preeclampsia diagnosis (orange triangles), or delivery (purple triangles) are indicated for individual preeclamptic patients (orange lines) and controls (purple lines). PBMCs were either left unstimulated or stimulated with a cocktail of LPS and IFN-α. Immune cells were barcoded, stained with surface and intracellular antibodies and analyzed with mass cytometry. The assay produced three categories of immune features, providing information about cell frequency (Fq) measured in 21 immune cell subsets (blue bar), basal intracellular signaling activity (green bar), and cell type-specific signaling capacity in response to stimulation with LPS and IFN-α (red bar). The number of immune features contained within each data category is indicated in parentheses. Correlation network reveals the relationships between immune features within and across mass cytometry data categories. A correlation network highlights the relationship between measured immune features (Spearman's coefficient).

Peripheral blood mononuclear cells (PBMCs) were prepared and cryopreserved according to standard protocols. On the day of sample stimulation, PBMCs at indicated time points (Figure 1) were thawed and rested in culture media containing 10% fetal bovine serum (Gibco) at 37°C for 2 h. PBMCs were counted and checked for viability.

PBMC samples were stimulated with either lipopolysaccharride (LPS) (1 μg/mL) and interferon-α (IFN-α) (100 ng/mL), or left unstimulated at 37°C for 15 min, then fixed for further analysis with mass cytometry. The rationale for choosing the combined stimulation condition LPS+IFN-α in this study was driven by results from the multivariate model of normal pregnancy (26) and by experimental constraints. Of the 4 stimulation conditions used in our previous study, the basal (unstimulated), LPS and IFN-α stimulated conditions provided the most information to the multivariate model of normal pregnancy (basal, LPS, and IFN-α and IL-2+IL-6 stimulations accounted for 25, 46, 17, and 12% of the model features, respectively). To maximize the information obtained from stimulated samples, LPS and IFN-α were utilized together after ensuring that little overlap was detected in our immunoassay between immune signaling responses to LPS (restricted to pERK1/2, pP38, pMAPKAPK2, pS6, pCREB, pNF-κB in innate immune cells) and to IFN-α (restricted to pSTAT1, pSTAT3, pSTAT5, pSTAT6 in innate and adaptive immune cells) (Figure S2).

### Sample Barcoding and Minimization of Experimental Batch Effect

To minimize the effect of experimental variability on mass cytometry measurements between samples from different time points and between samples from the control and preeclampsia groups, samples corresponding from the entire time series collected from one woman with preeclampsia and one control were processed, barcoded, pooled, stained and run simultaneously on the mass cytometry instrument (29, 30). There was no difference in total cell count, live cell count, viability, and storage time at time point 1 or 2 between the two groups.

### Antibody Staining and Mass Cytometry

The mass cytometry antibody panel included 22 antibodies that were used for phenotyping of immune cell subsets and 11 antibodies for the functional characterization of immune cell responses (Table S1). Antibodies were either obtained pre-conjugated (Fluidigm, Inc.) or were obtained as purified, carrier-free (no BSA, gelatin) versions, which were then conjugated in-house with trivalent metal isotopes utilizing the MaxPAR antibody conjugation kit (Fluidigm, Inc.). After incubation with Fc block (Biolegend), pooled barcoded cells were stained with surface antibodies then permeabilized with methanol and stained with intracellular antibodies. All antibodies used in the analysis were titrated and validated on samples that were processed identically to the samples used in the study. Barcoded and antibody-stained cells were analyzed on a Helios mass cytometer (Fluidigm, Inc.).

### Derivation of Immune Features

The mass cytometry data was normalized using Normalizer v0.1 MATLAB Compiler Runtime (MathWorks) (31). Files were then de-barcoded with a single-cell MATLAB de-barcoding tool (30). Manual gating was performed using CellEngine (https://immuneatlas.org/#/) (Primity Bio, Fremont, CA) according to our previous gating strategy (Figure S1) (26). The following 21 cell types were included in the analysis: B cells, Natural Killer cells (NK), CD56^hi^CD16^−^ NK cells, CD56^lo^CD16^+^ NK cells, CD4^+^T cells, CD4^+^CD45RA^−^T cells (CD4^+^Tmem), CD4^+^CD45RA^+^T cells (CD4^+^Tnaive), CD4^+^Tbet^+^T cells (Th1), CD25^+^FoxP3^+^CD4^+^T cells (Tregs), CD8^+^T cells, CD8^+^CD45RA^−^T cells (CD8^+^Tmem), CD8^+^CD45RA^+^T cells (CD8^+^Tnaive), CD8^+^Tbet^+^CD45RA^−^ cells, CD8^+^Tbet^+^CD45RA^+^T cells, TCR_γδ_T cells, CD14^+^CD16^−^ classical monocytes (cMCs), CD14^−^CD16^+^ non-classical MCs (ncMCs), CD14^+^CD16^+^ intermediate MCs (intMCs), monocytic myeloid-derived suppressor cells (M-MDSCs), myeloid dendritic cells (mDCs), and plasmacytoid dendritic cells (pDCs).

#### Cell frequency features

Cell frequencies were expressed as a percentage of gated singlet live mononuclear cells (cPARP^−^CD45^+^CD66^−^).

#### Basal signaling immune features

Basal intracellular signaling activities were derived from the analysis of unstimulated samples. The phospho-signal intensity of the following functional markers was simultaneously quantified per single cells: pSTAT1, pSTAT3, pSTAT5, pSTAT6, pNFκB, pMAPKAPK2, pP38, prpS6, pERK1/2, pCREB. Total IκB was measured to assess IκB degradation. For each cell type, signaling immune features were calculated as the mean signal intensity (arcsinh transformed value) of each signaling protein.

#### Intracellular signaling response features

For each cell type, the arcsinh difference (arcsinh ratio) in signal intensity between the stimulated and unstimulated conditions was calculated for each functional marker. Stimulation conditions that yielded little or no responses in optimization experiments (Figure S2) were excluded from the analysis.

### Correlation Network

Spearman correlation analyses were performed between pairs of immune features measured at each time point. The graphical representation of the correlation network shows edges for significant correlations between data pairs (*p* < 1.0E-12). Edge length is proportional to –log10 (*p*-value). The graph layout was calculated using the t-SNE algorithm and visualized using the i-graph R package (32). Communities of correlated immune features were detected by multi-level modularity optimization algorithm using the “cluster_louvain” function from i-graph R package (33, 34).

### Parametrization of Immune Feature Dynamics

Parametrization of immune feature dynamics: For each immune feature, the rate of change between the two sampling time points was estimated as:

$$\begin{array}{l} \rho \;=\;\frac{{immune\;feature}_{T2}-{immune\;feature}_{T1}}{GA_{T2}-GA_{T1}} \end{array}$$

### Statistical Analyses

A multivariate LASSO (least absolute shrinkage and selection operator) linear logistic regression method was utilized for this study (35). This method was chosen as it uses an *L*_1_ penalization over the classifier's coefficients to develop a “sparse” model that is suitable for the modular and correlated structure of the immune dataset (35). In addition, the LASSO method utilizes few free-parameters, which enables effective optimization using cross-validation.

The feature matrix was constructed using the rate of change of immune feature (ρ) as follows: For a design matrix *P* of immune feature rates (ρ), and a binary response vector of preeclampsia Y, a multivariate linear logistic LASSO regression model was developed to calculate the coefficients β for each entity in *P* to maximize the overall log-likelihood using the conditional likelihood of C given P,

$$\begin{array}{l} l\left(\beta \right)=\;\overset{n}{\underset{i=1}{\sum }}log\;p_{yi}\left(\rho_{i\;};\beta \right) \end{array}$$

where

$$\begin{array}{l} p_{yi}\left(\rho_{i\;};\;\beta \right)=\mathrm{Pr}\left(C=\;y_{i}|P=\;\rho_{i\;};\beta \right) \end{array}$$

With this convention, log-likelihood can be rewritten as

$$\begin{array}{l} l\left(\beta \right)=\;\overset{n}{\underset{i=1}{\sum }}\left[y_{i}\beta^{T}\rho_{i\;}-log\left(1+\exp\left(\beta^{T}\rho_{i\;}\right)\right)\right] \end{array}$$

An *L*_1_ regularization was applied on the β coefficient to reduce the model complexity, such that

$$\begin{array}{l} l\left(\beta \right)=\;\overset{n}{\underset{i=1}{\sum }}\left[y_{i}\beta^{T}\rho_{i\;}-\log\left(1+\exp\left(\beta^{T}\rho_{i\;}\right)\right)\right]+\lambda \overset{p}{\underset{j=1}{\sum }}|\beta_{j}| \end{array}$$

where lambda λ is selected by cross-validation. This produces a sparse model in which only a limited number of features are used (35).

The model was trained on 20 randomly-selected patients and tested on the remaining three. After 100 iterations, the mean of all predictions for a given patient in the test set was used as the final blinded prediction. This strategy minimizes the risk of overfitting by ensuring the models are always tested on samples that were not previously seen by the algorithm.

#### Model Reduction

The relative weights of immune features selected by the LASSO method were determined using the frequency at which individual immune features were selected through all cross-validation iterations. The top ten features were chosen by a piecewise regression model, a statistical technique used to specify an abrupt shift over the response variable corresponding to the explanatory variable.

We used student *t*-test to compare individual immune features between control and preeclampsia groups if the data is normal distributed test by Shapiro-Wilk test, otherwise, a Mann-Whitney nonparametric test was used.

### Confounder Analysis

We analyzed 14 demographic and comorbid conditions, including age, race, ethnicity, Body Mass Index (BMI), GA at delivery, total number of pregnancies, multiparity, parity, gestational diabetes, type 2 diabetes, preeclampsia history, autoimmune disease, and chronic hypertension. Women with preeclampsia had higher BMI (*p* = 2.0E-3) than the controls (student *t*-test). Higher rates of type 2 diabetes (*p* = 4.5E-2), chronic hypertension (*p* = 4.5E-2) and autoimmune diseases (*p* = 8.0E-3) were found in the patients with preeclampsia, which were significant by Fisher's exact test. To test whether these four comorbidities had an effect on immune features associated with preeclampsia, multiple linear regression analyses were performed to determine whether preeclampsia is a significant predictor of each immune feature when accounting for the four relevant comorbidities. This confounder analysis was performed using SPSS version 12.0 (SPSS Inc., Chicago, IL, USA).

## Results

### Study Cohort

The 11 study participants with preeclampsia were slightly younger and heavier than the 12 study participants from the control group (Table 1). Seven of the women with preeclampsia had severe features, and two had early-onset preeclampsia (including one patient with severe features). Participants with preeclampsia had more comorbidities, including arterial hypertension, diabetes, and autoimmune diseases, including systemic lupus erythematosus (SLE) (Table 1). Samples were collected well before clinical diagnosis of preeclampsia: a median of 13 weeks (interquartile range (IQR), 12 to 14). The gestational age (GA) at time of sampling did not differ between the two groups (median at the first time point (T1): 11 ± 1.8 weeks vs. 11 ± 2.0 weeks, *p* = 0.48; median at the second time point (T2): 25.5 ± 4.2 weeks vs. 25 ± 4.2 weeks, *p* = 0.36).

### Deep Profiling of Maternal Immune Responses in Healthy and Preeclamptic Pregnancies

PBMCs collected longitudinally during pregnancy were analyzed using a 41-parameter immunoassay for an in-depth profiling of peripheral immune cell adaptations. For each patient sample, 371 immune features were quantified on a per cell basis in 21 distinct innate and adaptive immune cell subsets (Figure 1). Immune features included cell frequencies and the activity (e.g., phosphorylation state) of 11 intracellular signaling proteins measured at baseline (basal signaling activity) as well as in response to extracellular stimulations with IFN-α and LPS (Figure 1; Figure S2). Stimulation conditions were chosen to activate receptor-specific signaling responses (Toll-Like Receptor (TLR) 4-dependent signaling for LPS, Janus kinase (JAK)-Signal Transducer and Activator of Transcription (STAT) signaling for IFN-α) that were most informative in characterizing immune cell dynamics in our previous mass cytometry analyses of healthy pregnancies (26).

**Figure 2:**
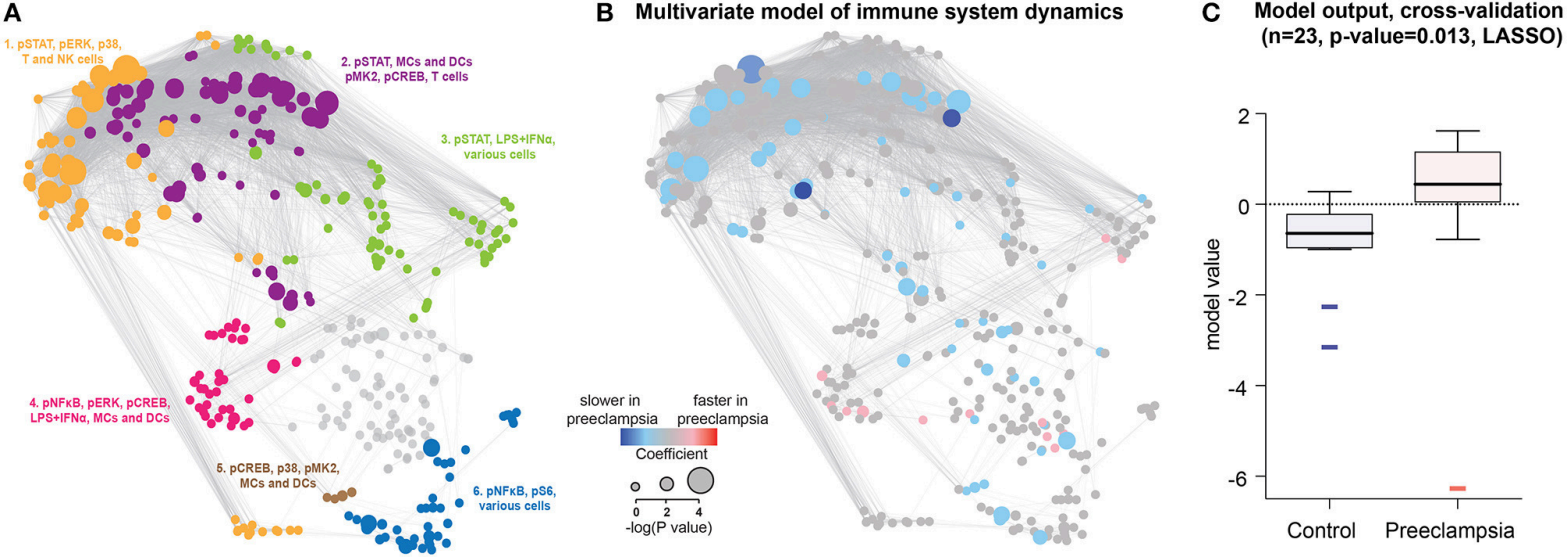
Predictive modeling of immune response dynamics associated with preeclampsia. **(A)** The correlation network segregates into 6 major communities of correlated immune features. Communities were detected using the Louvain multi-level modularity optimization method (33, 34) and annotated on the basis of immune feature characteristics (signaling property, stimulation, or cell subset) most commonly represented within each community. **(B)** A predictive multivariate model built on immune feature dynamics (rate of change between the first and second time points). LASSO identified patients that develop preeclampsia within 12–14 weeks after the last sampling time. Red/blue dots highlight immune features that evolve faster/slower in preeclampsia compared to Control. Dot size indicates the –*log*_*10*_ of *p*-value of model components compared between preeclamptic women and controls (Student *t*-test). **(C)** Boxplots showing model prediction for controls and preeclamptic women (AUC 0.803, cross-validation *p*-value = 0.013).

The high-parameter immunological dataset yielded a correlation network that emphasized the interconnectivity of immune responses during pregnancy (Figure 1). The correlation network segregated into 6 major communities of closely interconnected immune features, which were identified using a multi-level modularity optimization algorithm (33, 34). These statistically defined communities were annotated on the basis of immune feature characteristics (signaling property, stimulation, or cell subset) most commonly represented within each community (Figure 2A).

In the control group, a targeted examination of select communities revealed peripheral immune cell adaptations that dovetailed with prior immune profiling studies of normal pregnancy (26, 36, 37). For instance, one of the communities (Community 1) was primarily defined by the basal activity of the transcription factor STAT5ab (phospho, pSTAT5 signal) in CD4^+^T cells (Figure S3A), which increased during pregnancy as previously reported (26). Another community (Community 3) contained features that pointed at increasing pSTAT1 responses to stimulation in NK cells during pregnancy (Figure S3B), consistent with prior *in vitro* and *in vivo* studies showing that NK cell-mediated pathogen responses are exacerbated during pregnancy (26, 36, 38). In addition, Treg cell frequency increased between the first and second trimesters of pregnancy (Figure S3C), consistent with prior reports of Treg dynamics during pregnancy (26, 39). Thus, the immunoassay utilized in this study was sensitive to detect established hallmarks of maternal immune adaptations during a normal pregnancy.

### Immune System-Wide Dynamics Are Disrupted in Preeclampsia

A number of observations in humans support the assessment of immune cell responses over time, rather than a cross-sectional assessment at a given time point, to understand how the human immune system adapts to a physiological or a pathological perturbation (40). We reasoned that an analysis focused on immune response dynamics would be particularly adapted to detect immune dysfunctions preceding the onset of preeclampsia.

To parameterize the dynamic changes in the peripheral immune system during pregnancy, the rate of change between the first and second sampling time points was calculated for each immune feature. The least absolute shrinkage and selection operator (LASSO) method (35) was applied to the dataset of immune feature dynamics. The predictive analysis identified a multivariate model that accurately differentiated women who developed preeclampsia from controls (Figures 2B,C). Components of the LASSO model were visualized on the correlation network as red or blue nodes highlighting immune features with accelerated or decelerated, respectively, dynamics in women who will develop preeclampsia (Figure 2B). The generalizability of the model was established using a stringent cross-validation method that accounts for the high-dimensionality of the dataset. No significant association was found between the LASSO model prediction and the presence of comorbid conditions, including autoimmune diseases, gestational diabetes, chronic hypertension and body mass index (BMI). When excluding patients with autoimmune disease and gestational diabetes, the LASSO model remained highly significant (cross-validated *p*-value = 0.016) and robust (AUC = 0.83). The results suggest that specific aspects of peripheral immune system dynamics, detectable 12–14 weeks before the clinical diagnosis of preeclampsia, are disrupted in preeclamptic pregnancies.

### Pro-inflammatory Immune Responses Early in Pregnancy Contribute to Abnormal Immune System Dynamics in Preeclampsia

**Figure 3:**
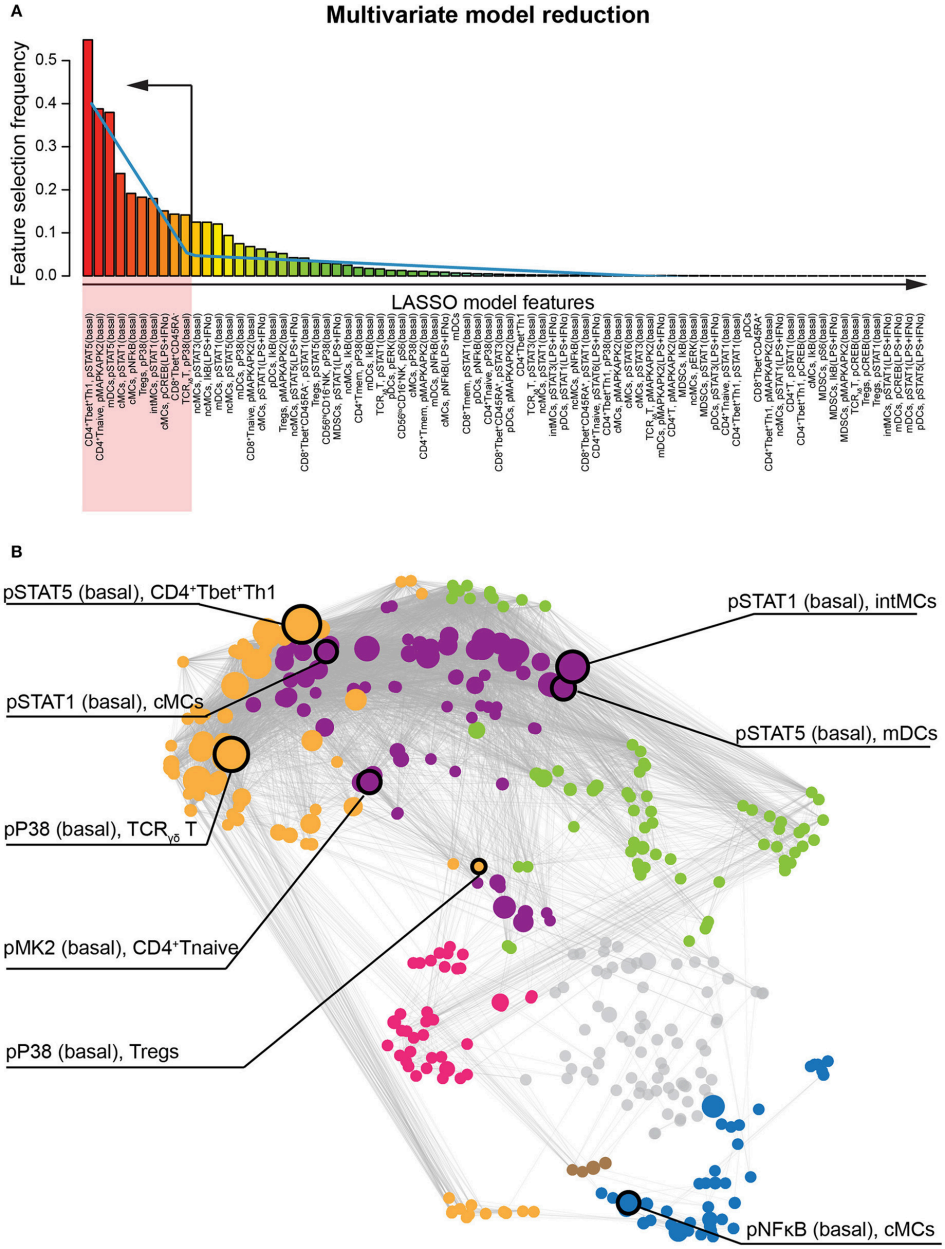
Identification of the most informative features classifying patients who develop preeclampsia. **(A)** The bar graph depicts the frequency of immune feature selection across all cross-validation iterations. Blue line indicates piecewise regression fit for identification of a breakpoint indicating ten immune features that are most informative to the multivariate LASSO model. **(B)** The most informative immune features and their respective immunological communities are highlighted on the correlation network.

The LASSO method allowed a system-level analysis of immune dysfunction in preeclampsia anchored by a statistically-stringent multivariate model. To highlight the most informative features of the multivariate model and facilitate biological interpretation, we applied a piecewise regression method that reduced the model to 10 components (Figure 3A) that were highly discriminating between control and preeclamptic pregnancies: the median area under the curve (AUC) was 0.90, within an IQR of 0.81 to 0.92. Most (90%) of the informative immune features were intracellular signaling responses (AUC 0.80-0.92), while the cell frequency features had a much weaker predictive performance for preeclampsia (AUC = 0.65). Eight out of these ten immune features remained highly significant as predictors of preeclampsia after controlling for demographic and clinical variables (BMI, presence of autoimmune disease, hypertension, and type 2 diabetes) in a multivariate linear regression analysis (Table S2).

**Figure 4:**
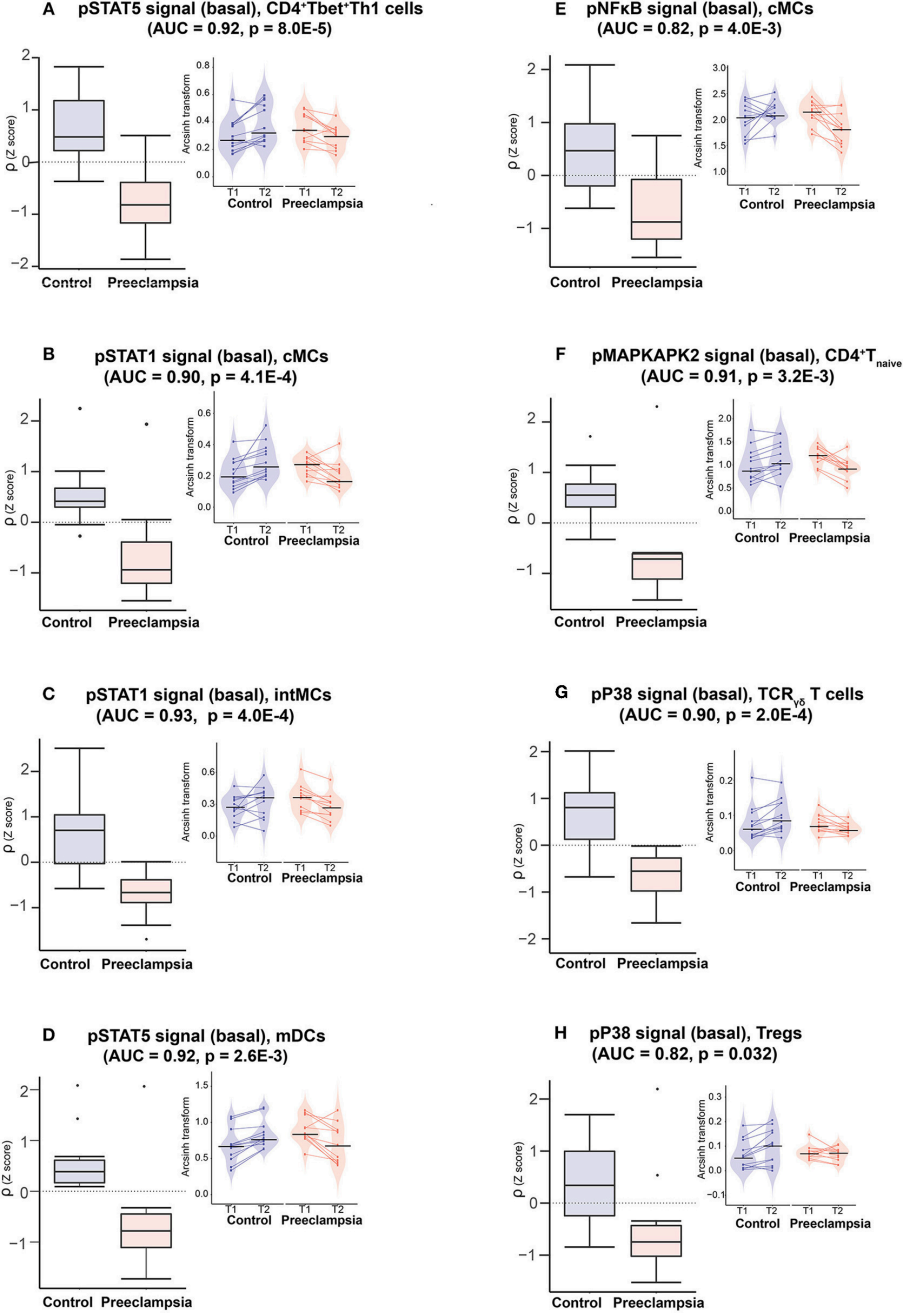
Model components reveals disrupted innate and adaptive immune cell dynamics in preeclampsia. Boxplots (**left panels**) depict the rate of change (ρ) of indicated immune feature for the eigth most informative model components. AUC and *p*-values are indicated on each graph (ROC analysis). Insets (**right panels**) depict immune feature values (arcsinh transform of the mass cytometry intracellular signal mean intensity) at individual time points (T1, T2) and for each patient. Color code: purple = controls, orange = preeclampsia.

These eight informative immune features appeared within 3 of the 6 communities, consistent with dysregulated immune dynamics that spanned multiple immune compartments (Figure 3B). The most informative feature was the pSTAT5 signal (basal) in CD4^+^Tbet^+^Th1 cells (AUC = 0.92, *p* = 8.0E-5, Figure 4A). The pSTAT5 signal increased consistently in Th1 cells between the first and second trimesters in the control group but decreased consistently in women who developed preeclampsia (Figure 4A). Further examination of individual time points revealed that the pSTAT5 signal in Th1 cells was higher in women with preeclampsia compared with controls in the first trimester and then gradually decreased during the second trimester (Figure 4A, inset). These results are consistent with the prevailing theory of the presence of a predominance of Th1 in preeclampsia (41), given that STAT5 can potentiate Th1 differentiation (42).

Because the JAK/STAT5 signaling pathway is implicated in multiple aspects of CD4^+^T cell differentiation, notably in the differentiation and stability of peripheral Tregs (43–45), we tested whether observed pSTAT5 dynamics were restricted only to Th1 cells. In Community 1 (defined by the STAT5 signaling response) the abnormal pSTAT5 dynamics were shared among several T cell subsets including CD4^+^ Tnaive cells and CD25^+^FoxP3^+^ Tregs (Figure S4). The pSTAT5 signal in Tregs increased between the first and second trimesters in the control group, but did not change in the preeclamptic women. The results highlight an important role for STAT5-dependent responses across multiple CD4^+^T cell subsets that are disrupted prior to the clinical onset of preeclampsia.

The remaining immune features of the reduced model suggested that, overall, strong pro-inflammatory cell responses early during pregnancy altered the immunologic trajectory of women who went on to develop preeclampsia. In innate immune compartments, the pSTAT1, pSTAT5, and pNFκB signals (basal) were elevated in intermediate monocytes (intMCs) (AUC = 0.93, *p* = 4.0E-4), myeloid dendritic cells (mDCs) (AUC = 0.92, *p* = 0.0026), and classical monocytes (cMCs) (pSTAT1: AUC = 0.90, *p* = 4.1E-4; pNFκB: AUC = 0.82, *p* = 4.0E-3), respectively, during the first trimester in preeclamptic women compared with controls (Figures 4B–E). These responses gradually decreased during preeclamptic pregnancies, but increased in the control group. In adaptive immune compartments, elevated pro-inflammatory signaling responses in Th1 cells (pSTAT5) during the first trimester of pregnancy were coupled with abnormal signaling dynamics in CD4^+^Tnaïve cells (pMAPKAPK2, AUC = 0.91, *p* = 3.2E-3), TCR_γδ_ (pP38, AUC = 0.9, *p* = 2.0E-4) and CD25^+^FoxP3^+^Tregs (pP38, AUC = 0.82, *p* = 0.032) (Figures 4F–H). Of note, the pP38 signal, which is required for Treg suppressive function (46), increased in Tregs during pregnancy in controls but not in women with preeclampsia.

## Discussion

We employed a high-parameter mass cytometry immunoassay for an in-depth assessment of the dynamics of the peripheral immune system during normal and preeclamptic pregnancies. Analysis of the high-dimensional immunological dataset identified immune system dysfunction detectable in the maternal blood 12–14 weeks before the clinical signs of preeclampsia were evident. Individual components of the multivariate model highlighted profound dysregulation of intracellular signaling dynamics that were strongly associated with the subsequent development of preeclampsia (median AUC 0.91, IQR [0.82, 0.92]).

High-parameter flow cytometry technologies such as mass cytometry have transformed the ability to profile the human immune system. However, the high dimensionality of the resulting data presents a major analytical challenge to conventional statistical analysis (47). Application of regularized regression algorithms (such as LASSO) combined with a cross-validation method to ensure generalizability of the model outputs provided a robust statistical solution to this analytic challenge (48). In this study, the LASSO analysis provided a statistically stringent multivariate model that distinguished healthy pregnancies from those with preeclampsia, while simultaneously assessing over 370 immune features. The performance of individual model components in stratifying women who develop preeclampsia was also remarkable. Using the AUC as a metric, the individual performances of the top five model components to predict preeclampsia were each above 0.9, which signifies excellent predictive performance. These results may be due to several aspects of our analysis that differ from prior studies reporting on immunological biomarkers of preeclampsia (6, 7, 18–20, 49). The functional interrogation of signaling responses, rather than cell distribution may have been more informative; the simultaneous survey of multiple innate and adaptive immune cell subsets allowed for agnostic identification of the most informative immune features; and the analysis, which focused on immune cell dynamics rather than static immunologic events, may have allowed a more sensitive detection of pregnancy-related immune dysfunctions.

The most informative features of our analysis were the basal pSTAT5 signals in CD4^+^T cell subsets (AUC = 0.92, *p* = 8.0E-5). Notably, the pSTAT5 signal in CD4^+^T cells was also the most informative component of a multivariate model predictive of the age of gestation in a prior study of normal pregnancy (26, 27). These findings, derived from two independent studies, suggest that assessing pSTAT5 dynamics in CD4^+^T cell subsets early in pregnancy may be a key feature of an immuno-assay predicting the risk for developing preeclampsia.

The JAK/STAT5 pathway has been implicated in multiple, and seemingly conflicting, aspects of CD4^+^T cell development. Downstream of IL-2, *in vivo* and *in vitro* studies show that the IL-2/STAT5 pathway controls Th2 differentiation [by inducing the expression of the IL-4 receptor (50)] as well as Th1 differentiation [by inducing the expression of Tbet and the IL-12 receptor (42)]. The IL-2/STAT5 pathway is also critical for promoting peripheral Tregs (44) and inhibiting Th17 differentiation (51). Therefore, the decreasing pSTAT5 signal—which was higher during the first trimester in the preeclamptic group than in the control group—observed in CD4^+^T cell subsets early in preeclamptic pregnancies likely reflects multiple dysfunctional processes affecting T cell differentiation, including increased Th1 over Th2 differentiation during the first trimester as well as decreased Treg differentiation between the first and second trimesters.

However, IL-2 is not the only factor regulating the JAK/STAT5 signaling pathway. In fact, multiple inflammatory, but also hormonal, and placental factors implicated in pregnancy converge onto the JAK/STAT5 pathway, including prolactin, chorionic somatomammotropin hormone (CSH)-1, IFN-γ and IL-3 (52, 53). Leptin, which has consistently been associated with preeclampsia in multiple large-scale proteomics studies can also activate the JAK/STAT5 pathway (54). Assessment of JAK/STAT5 signaling dynamics may therefore provide a sensitive cellular readout of immune dysfunction that reflects the integration of multiple signals ultimately driving abnormal CD4^+^T cell responses early in the pathogenesis of preeclampsia.

Several other results of our analysis resonated well with prior knowledge of immune system dysfunction associated with preeclampsia, notably among innate immune cell subsets. In general, observed differences in the rate of change of innate immune responses (decelerated in the preeclampsia group compared to the control group) were driven in part by higher signaling responses during the first trimester of pregnancy in the preeclampsia group. In cMCs, the basal pNFκB signals was increased in the preeclampsia group in the first trimester compared to the control group (Figure 4E). A similar finding was observed for the pSTAT1 signal in the pro-inflammatory intMCs monocyte subtype (Figure 4C). An elevated signaling activity in innate immune cells early during pregnancy is consistent with previous studies suggesting exaggerated activation of proinflammatory innate immune responses in patients who develop preeclampsia (55, 56).

Interestingly, the majority of dysregulated immune responses were signaling responses rather than frequency changes. In this regard, some of our findings differ from previous analyses of peripheral immune responses associated with preeclampsia. For instance, neither the frequency of Tregs or of Th1 cells (13, 14, 41)—which have previously been shown to differ between normal and preeclamptic pregnancies—were selected as informative features of the multivariate model. Instead, measurement over time of signaling responses in Tregs (pP38 and pSTAT5) and in CD4^+^Tbet^+^Th1 cells (pSTAT5) were among the strongest individual classifiers for preeclampsia. These results suggest that a functional read-out of proximal signaling responses may be more informative than the assessment of cell distribution alone in identifying immune dysfunction associated with preeclampsia.

This study has several limitations. The recruitment of study participants at a single hospital limits the generalizability of the results. Larger, multicenter studies will be required to generalize our findings to women from various demographic, ethnic, and socioeconomic backgrounds. The sample size was also too small to allow us to distinguish between early (GA < 34 weeks, *n* = 2) and late (*n* = 9) onset preeclampsia. Determining whether immune system dynamics differ between subtypes of preeclampsia will be important, since different pathophysiological mechanisms may underlie the clinical spectrum of preeclampsia. The study did not exclude patients with autoimmune diseases, such as SLE, which are known risk factors for preeclampsia (57–59). In particular, the pathogenesis of SLE involves an imbalance between Tregs and Th17 cells (60), which is also associated with immunological dysregulation in preeclampsia (13, 61, 62). Interestingly, our LASSO model and its major individual components remained strongly associated with preeclampsia when excluding patients with autoimmune diseases from our analysis. These results are in line with previous observations showing differential transcriptomic immune profiles in pregnant women with SLE who do or do not develop preeclampsia, suggesting that certain immune responses associated with preeclampsia are independent of SLE (63). While our study is underpowered to detect immune responses associated with SLE and other comorbidities (such as gestational diabetes), our results suggest that reported differences between the two study groups are not driven by the presence of these known immunological confounders.

In addition, while mass cytometry offers unprecedented informational content at the single-cell level, the technology remains limited to the measurement of ~50 pre-selected phenotypic and functional parameters per immune cell. For instance, selected antibody panel did not allow for the analysis of intracellular cytokines, which would be helpful for further characterization of Th1, Th2, and Th17 cell subsets. We cannot exclude that additional phenotypic markers not included in the current analysis will allow detecting more informative and predictive cell frequency features. Similarly, the choice of stimulation conditions was limited by sample availability. Finally, the analysis was limited to immune cell dynamics that vary linearly with time, and did not capture non-linear immunological adaptations which are known to occur during pregnancy. However, the approach provides an analytical and statistical framework for future studies aimed at exhaustive characterizations of immune cell dynamics in normal and preeclamptic pregnancies.

In summary, our study reveals significant alterations in the dynamics of maternal immune system adaptations months before the clinical onset of preeclampsia. The data and analytical approaches presented here suggest that measures of maternal immune system dynamics early in pregnancy hold significant promise for identifying women at risk for developing preeclampsia later during pregnancy.

## Data Availability

Raw data is publicly available at http://flowrepository.org under experiment ID FR-FCM-ZYRQ. Anonymous reviewer access is provided using the following links: http://flowrepository.org/id/FR-FCM-ZYRQ. Clinical annotations for each sample are provided as an attachment to the repository. Extracted features are available through: https://nalab.stanford.edu/wp-content/uploads/pbmcpedata.zip.

## Ethics Statement

This study was carried out in accordance with the recommendations of the Lucile Packard Children's Hospital at Stanford University Institutional Review Board with written informed consent from all subjects. All subjects gave written informed consent in accordance with the Declaration of Helsinki. The protocol was approved by the Stanford Institutional Review Board.

## Author Contributions

BG conceived and supervised the execution of the study and wrote the manuscript. BG supervised all mass cytometry data collection and curation. NA supervised all statistical analysis. MA assisted in interpreting data and writing the manuscript. XH conduct the sample process, cytometry data collection and analysis. MG performed statistical analysis. XH, MG, IAS, and LP wrote the manuscript. KA, RW, AJ, and DG assisted in clinical data collection and interpretation. EG and AST assisted in the fabrication of reagents and sample processing for mass cytometry analysis. JE, BB, ET, NS, AC, AT, JH, and RF assisted in data visualization. RW, VW, MD, YB, MH, CQ, RG, BC, GS, and DS assisted in interpreting mass cytometry data and writing the manuscript.

### Conflict of Interest Statement

The authors declare that the research was conducted in the absence of any commercial or financial relationships that could be construed as a potential conflict of interest.
