# Supplementary material for: Differential Dynamics of the Maternal Immune System in Healthy Pregnancy and Preeclampsia
Source: Front Immunol. 2019 Jun 11;10:1305. doi: 10.3389/fimmu.2019.01305 (PMC6584811; doi:10.3389/fimmu.2019.01305)
Supplement: Supplementary file 1 [file Data_Sheet_1.pdf]

Supplementary Material

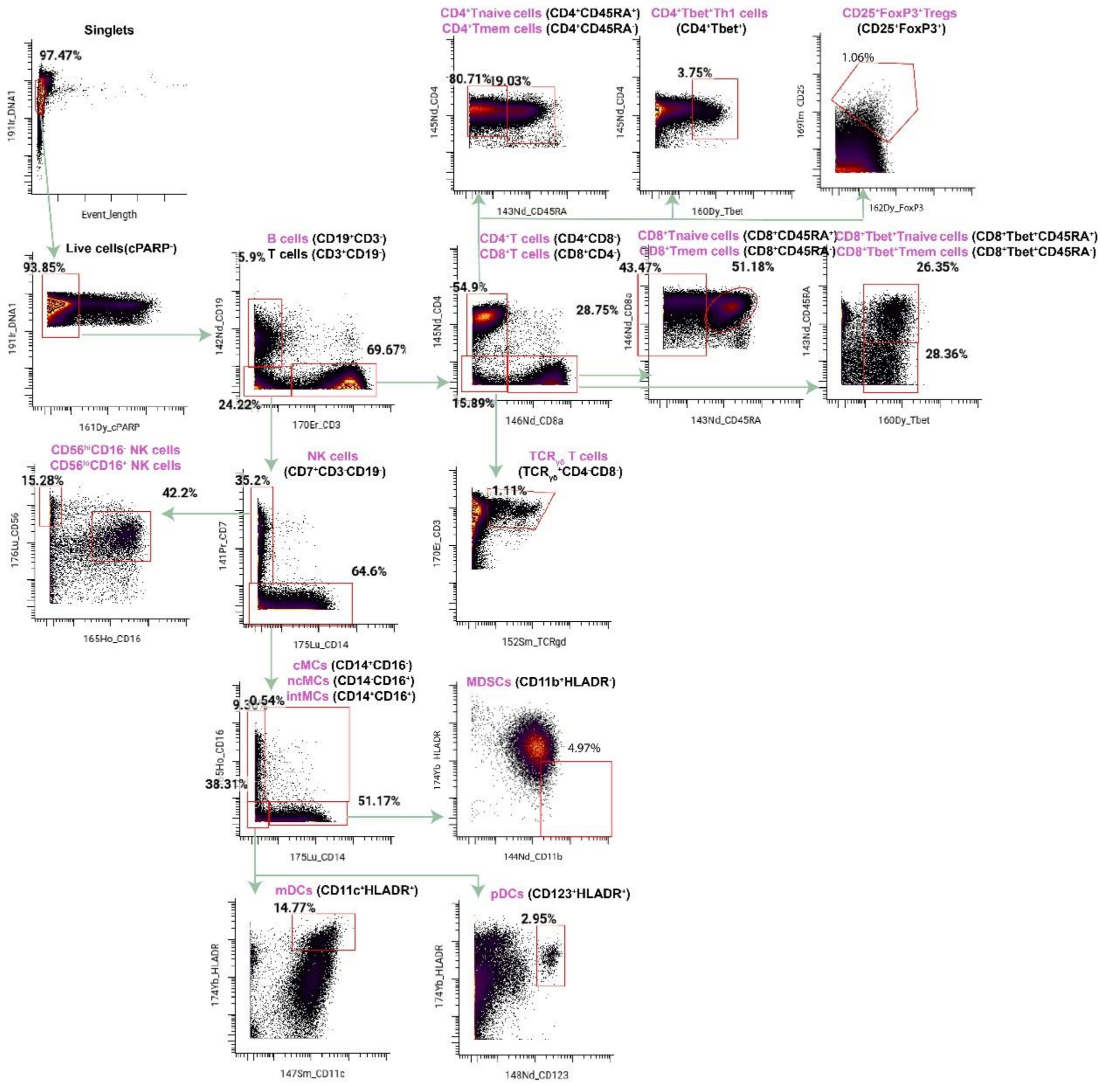

Figure S1. Gating strategy. Twenty-one immune cell subsets were gates based on established guidelines for manual gating of innate and adaptive cells *(57)*.

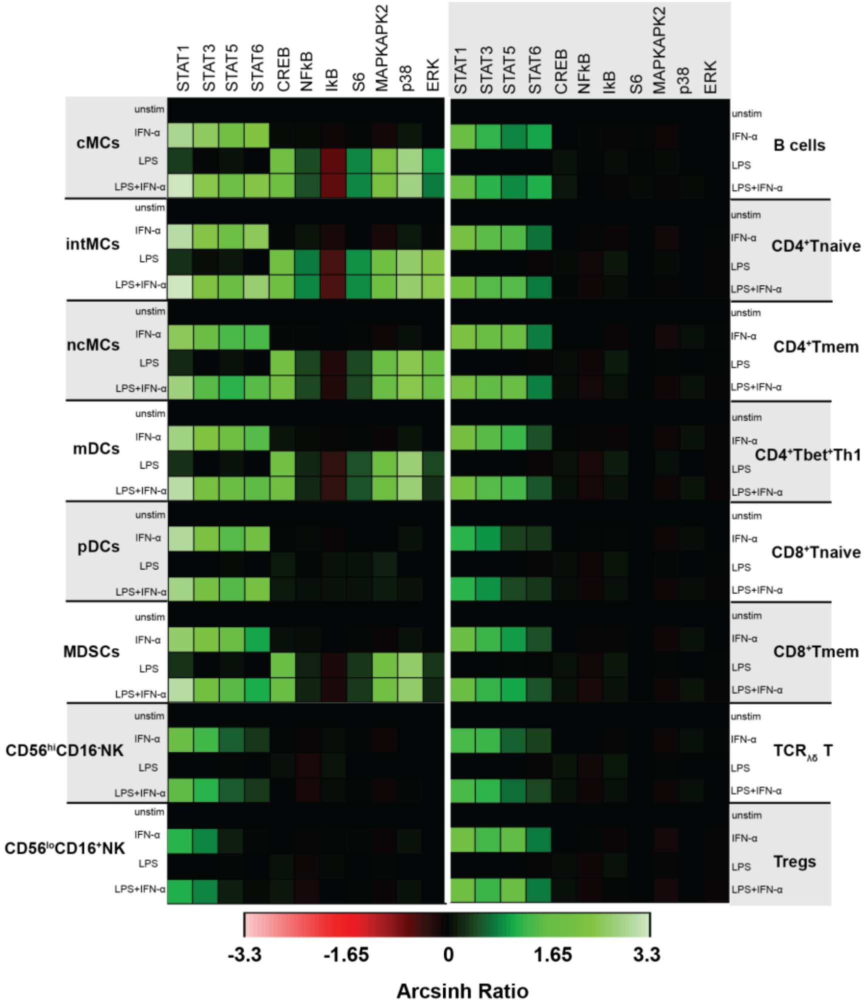

Figure S2. Optimization of the stimulation condition. PBMC sample availability allowed for only one stimulation to be performed. LPS and IFN-α were utilized in combination to maximize the functional information obtained from the stimulated samples. Blood samples from a healthy volunteer was left unstimulated (*first row*) or stimulated with LPS alone (1 μg/mL, *second row*), IFN-α alone (100 ng/mL, *third row*), or both (*fourth row*) for 15min and analyzed using mass cytometry. The heatmaps show that the mass cytometry immunoassay detected little overlap in immune cell signaling activities in response to LPS and IFN-α alone. Immune signaling responses to LPS were restricted to pERK1/2, pP38, pMAPKAPK2, pS6, pCREB, pNF-κB and total IκB in innate immune cells. Immune signaling response to IFN-α were restricted to pSTAT1, pSTAT3. pSTAT5, pSTAT6 in innate and adaptive immune cells).

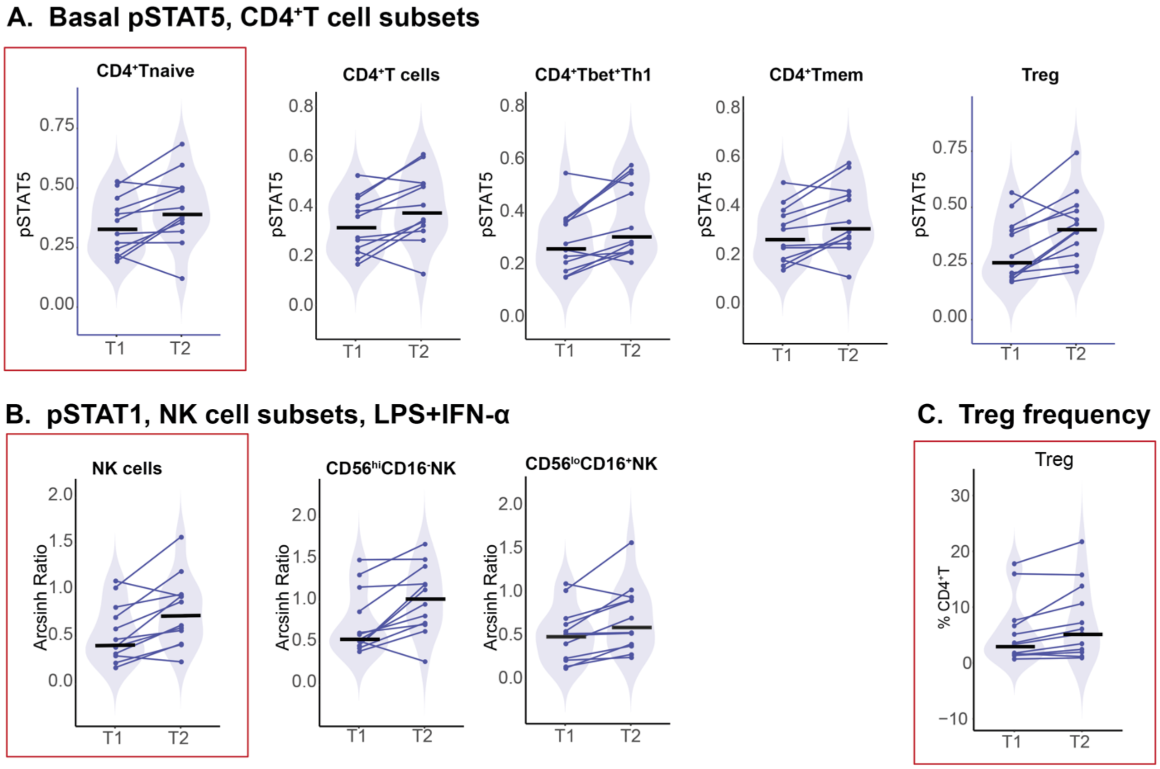

Figure S3. The mass cytometry immunoassay detects expected immune cell adaptations during a healthy pregnancy. Targeted analysis of select immune adaptations in the control group confirms previously reported signaling and frequency immune cell adaptations during a normal pregnancy, including (A) increased basal pSTAT5 signals in $CD4^{+}$Tnaive (p=0.015), $CD4^{+}$Tmem (p=0.013) and Treg cell subsets (p=0.008), (B) increased pSTAT1 response to LPS+IFN-α in NK cell subsets (p=0.004) and (C) increased Treg cell frequency (p=0.008).

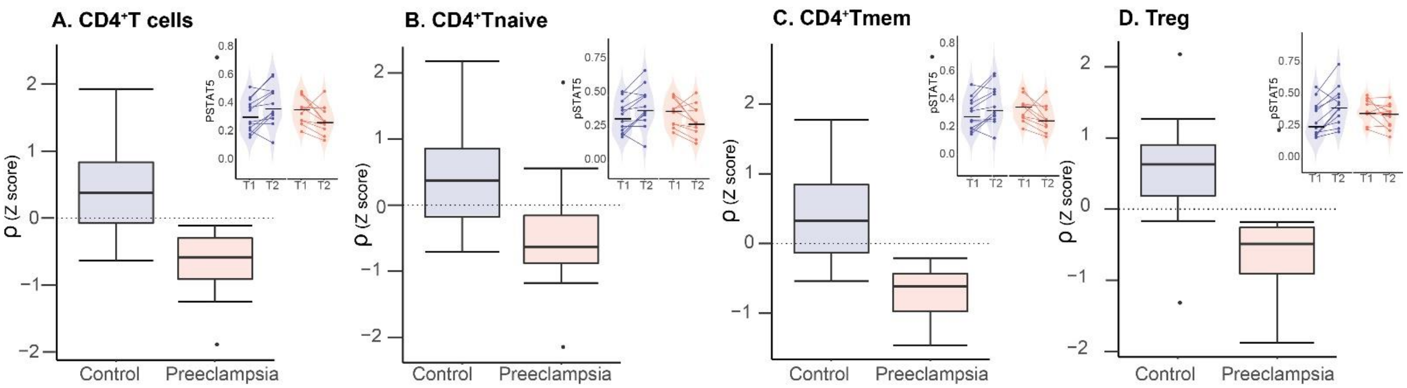

Figure S4. pSTAT5 signal (basal) in CD4$^+$T subsets. Total CD4$^+$T cells (A), CD4$^+$Tnaive (B), CD4$^+$Tmem (C), CD25$^+$FoxP3$^+$Tregs (D). Boxplots (left panel) depict immune feature rate of change (ρ) for controls (purple) and preeclampsia (orange) study participants. Insets (right panels): Immune feature values (arcsinh transform of mass cytometry intracellular signal mean intensity) are represented for individual time points and for each patient (lines).

Antibody	Manufacturer	Symbol	Mass	Clone	Comment
Barcode 1	Trace Sciences	Pd	102		Barcode
Barcode 2	Trace Sciences	Pd	104		Barcode
Barcode 3	Trace Sciences	Pd	105		Barcode
Barcode 4	Trace Sciences	Pd	106		Barcode
Barcode 5	Trace Sciences	Pd	108		Barcode
Barcode 6	Trace Sciences	Pd	110		Barcode
CD235ab	Biolegend	In	113	HIR2	Phenotype
CD61	BD	In	113	VI-PL2	Phenotype
CD45	Biolegend	In	115	HI30	Phenotype
CD66	BD	La	139	CD66a-B1.1	Phenotype
CD7	BD	Pr	141	M-T701	Phenotype
CD19	Biolegend	Nd	142	HIB19	Phenotype
CD45RA	Biolegend	Nd	143	HI100	Phenotype
CD11b	Fluidigm	Nd	144	ICRF44	Phenotype
CD4	Fluidigm	Nd	145	RPA-T4	Phenotype
CD8a	Fluidigm	Nd	146	RPA-T8	Phenotype
CD11c	Fluidigm	Sm	147	Bu15	Phenotype
CD123	Biolegend	Nd	148	6H6	Phenotype
pCREB (pS133)	CST	Sm	149	87G3	Function
pSTAT5 (pY694)	Fluidigm	Nd	150	47	Function
pP38 (pT180/pY182)	BD	Eu	151	36/p38	Function
TCRγδ	Fluidigm	Sm	152	11F2	Phenotype
pSTAT1 (pY701)	Fluidigm	Eu	153	58D6	Function
pSTAT3 (pY705)	CST	Sm	154	M9C6	Function
pS6 (pS235/pS236)	CST	Gd	155	D57.2.2E	Function
CD33	Fluidigm	Gd	158	WM53	Phenotype
pMAPKAPK2 (pT334)	Fluidigm	Tb	159	27B7	Function
Tbet	Fluidigm	Gd	160	4B10	Phenotype
cPARP	BD	Dy	161	F21-852	Function
FoxP3	Fluidigm	Dy	162	PCH101	Phenotype
IκB	Fluidigm	Dy	164	L35A5	Function
CD16	Fluidigm	Ho	165	3G8	Phenotype
pNFκB (pS529)	Fluidigm	Er	166	K10-895.12.50	Function
pSTAT6 (pY641)	Fluidigm	Er	168	18	Function
CD25	Biolegend	Tm	169	M-A251	Phenotype
CD3	Fluidigm	Er	170	UCHT1	Phenotype
pERK1/2 (pT202/pY204)	Fluidigm	Yb	171	D13.14.4E	Function
HLA-DR	Fluidigm	Yb	174	L243	Phenotype
CD14	Fluidigm	Yb	175	M5E2	Phenotype
CD56	BD	Yb	176	NCAM16.2	Phenotype
DNA1	Fluidigm	Ir	191		DNA
DNA2	Fluidigm	Ir	192		DNA

Table S1. Antibody panel used for mass cytometry analysis.

Features	Selection frequency	p-value	AUC	Community	p-value (adjusted for clinical covariates)
pSTAT5 (basal), $CD4^{+}Tbet^{+}Th1$	0.5483	0.0001	0.9167	1	0.001
MAPKAPK2 (basal), $CD4^{+}$Tnaive	0.3879	0.0032	0.9091	2	0.014
pSTAT5 (basal), mDCs	0.3798	0.0026	0.9167	2	0.003
pSTAT1 (basal), cMCs	0.2379	0.0117	0.7955	2	<0.001
pNFkB (basal), cMCs	0.1916	0.0040	0.8182	6	0.005
pP38 (basal), Tregs	0.1828	0.0323	0.8182	1	0.044
pSTAT1 (basal), intMCs	0.1796	0.0004	0.9318	2	0.001
pCREB (LPS+IFN-α), cMCs,	0.1510	0.0177	0.7955	4	0.259
$CD8^{+}Tbet^{+}CD45RA^{-}$, frequency	0.1435	0.1137	0.6591	N/A	0.755
pP38 (basal), $TCR_{\gamma\delta}T$,	0.1415	0.0002	0.9015	1	<0.001

Table S2. Reduced LASSO model components. As BMI, autoimmune disease, hypertension, type 2 diabetes were significantly different between the two study groups, a multiple linear regression was performed to determine whether the presence of preeclampsia was still an independent predictor of the components of the reduced LASSO model after accounting for these potential clinical confounders. Results showed that the presence of preeclampsia was still a significant predictor for eight out of the remaining nine model components after adjusting for BMI, autoimmune disease, hypertension, and type 2 diabetes. Preeclampsia was not a significant predictor of the pCREB, cMCs, LPS+IFN-α model component after accounting for these confounding variables (p-value > 0.05, Mann-Whitney U test).
